# Persistent fifth aortic arch associated with aortic coarctation: a case of surgical correction without artificial material

**DOI:** 10.1186/s13019-021-01664-y

**Published:** 2021-09-28

**Authors:** Chang Hun Kim, Hyungtae Kim, Kwang Ho Choi, Si Chan Sung, Hoon Ko, Ki Seok Choo

**Affiliations:** 1 Department of Thoracic and Cardiovascular Surgery, Research Institute for Convergence of Biomedical Science and Technology, Pusan National University Yangsan Hospital, Geumo-ro 20, Beomeo-ri, Mulgeum-eup, Yangsan-si, Gyeongsangnam-do 50612 Republic of Korea; 2 Department of Pediatrics, Research Institute for Convergence of Biomedical Science and Technology, Pusan National University Yangsan Hospital, Geumo-ro 20, Beomeo-ri, Mulgeum-eup, Yangsan-si, Gyeongsangnam-do 50612 Republic of Korea; 3 Department of Radiology, Research Institute for Convergence of Biomedical Science and Technology, Pusan National University Yangsan Hospital, Geumo-ro 20, Beomeo-ri, Mulgeum-eup, Yangsan-si, Gyeongsangnam-do 50612 Republic of Korea

**Keywords:** Persistent fifth aortic arch, Coarctation of the aorta, Congenital heart disease

## Abstract

**Background:**

Persistent fifth aortic arch (PFAA) is a rare anomaly often associated with aortic coarctation or interruption, and various surgical techniques for this anomaly have been reported. Herein, we show a case of an infant with PFAA and severe aortic coarctation.

**Case presentation:**

A 41-day-old female infant was admitted for sustained fever. Initially, the patient was diagnosed with bacterial meningitis, and echocardiography showed PFAA with severe aortic coarctation. Because the patient presented progressive oliguria and metabolic acidosis, she was transferred for emergency cardiac surgical intervention. The aortic arch was reconstructed using end-to-side anastomosis between the fifth aortic arch and the descending aorta without any artificial conduit or patching material.

**Conclusions:**

PFAA with aortic coarctation can be repaired by various surgical methods. Among them, our surgical approach is easy and effective, has growth potential, and an additional surgery is not needed.

**Supplementary Information:**

The online version contains supplementary material available at 10.1186/s13019-021-01664-y.

## Background

Persistent fifth aortic arch (PFAA) is a rare congenital cardiac malformation frequently associated with aortic coarctation or interruption [4]. Various surgical repair techniques for PFAA have been reported [1–6]. Herein, we show a case of an infant with PFAA and severe aortic coarctation, which was successfully repaired without any artificial conduits or patching material.

### Case presentation

**Fig. 1:**
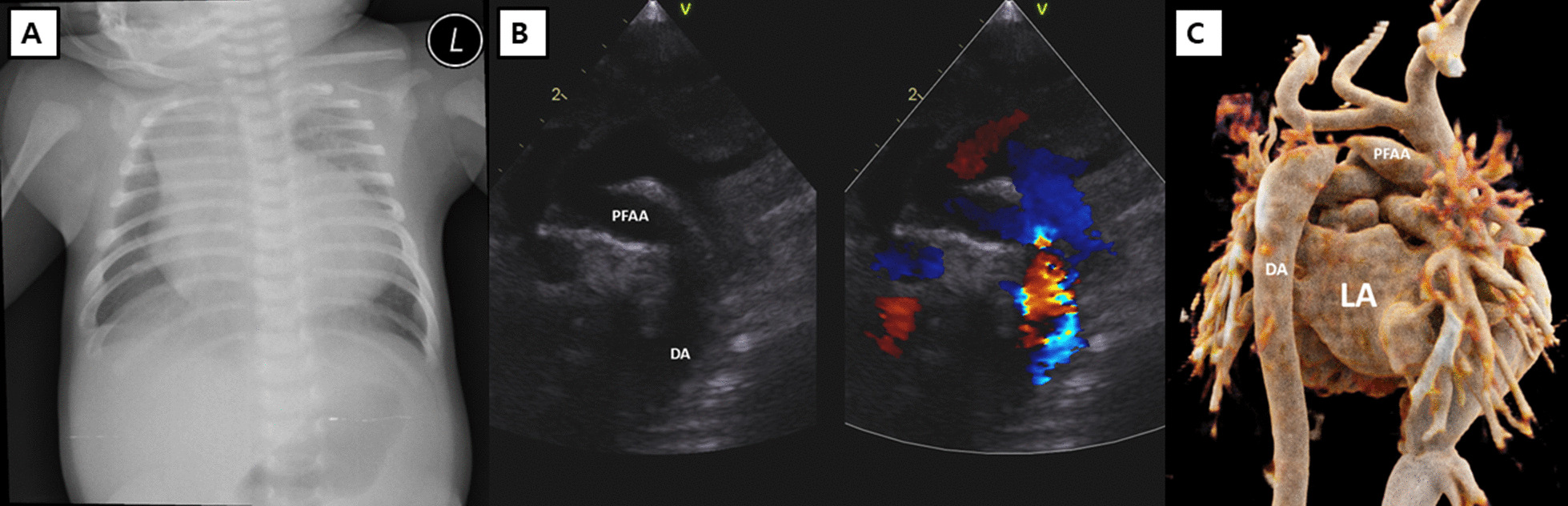
Severe cardiomegaly was identified on the initial chest x-ray (**a**), and preoperative echocardiography (**b**) and three-dimensional computed tomography angiography (posterior view) showed the persistent fifth aortic arch with severe aortic coarctation (**c**)

A 41-day-old female infant was admitted for sustained fever. The patient was diagnosed with bacterial meningitis by analysis of the cerebrospinal fluid and treated with intravenous antibiotics. During the hospital stay, the patient showed desaturation accompanied by cyanosis and cardiomegaly (cardiothoracic ratio, 0.77) on chest X-ray (Fig. 1a). Due to a constant increase in oxygen demand, the patient was intubated and transferred to the pediatric intensive care unit. Echocardiography showed coarctation of the aorta with decreased left ventricular function, atrial septal defect (ASD), and mitral valve regurgitation (Fig. 1b). Three-dimensional computed tomography (CT) angiography showed PFAA with aortic coarctation (Fig. 1c). Because the patient showed progressive oliguria and metabolic acidosis, we decided to perform emergency surgery.

**Fig. 2:**
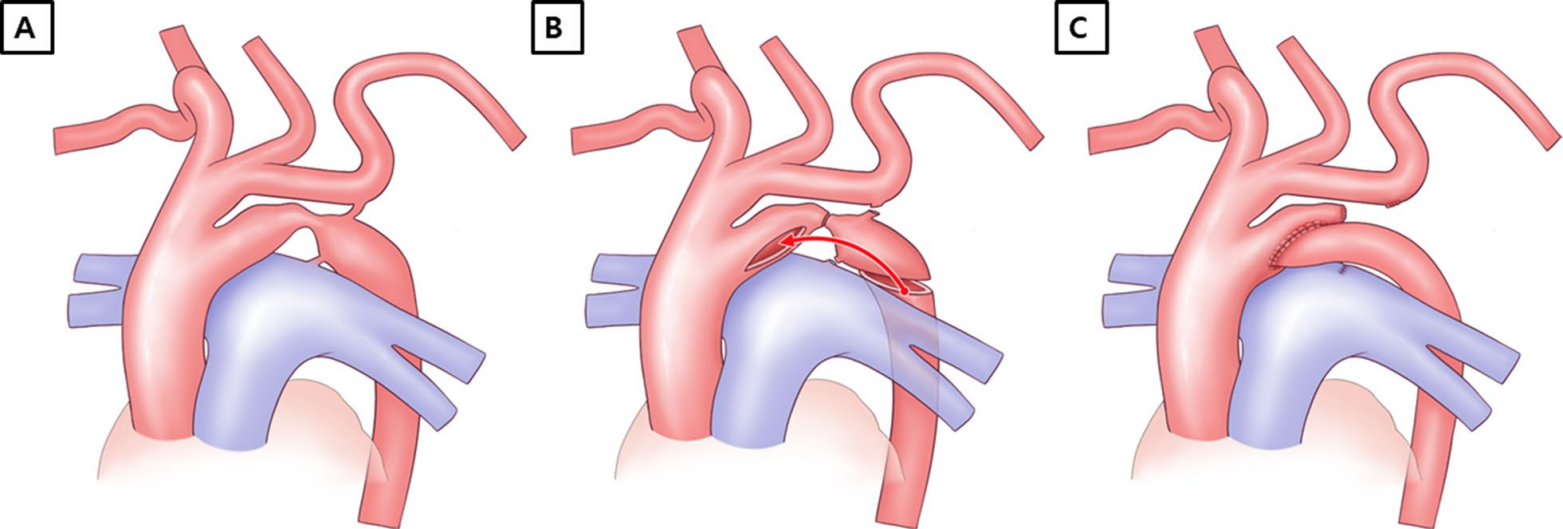
Schematic drawing of the operative technique. The anatomic morphology of the aortic arch (**a**) and after division of the isthmic portion and the ductus arteriosus (**b**). The proximal stump of the fifth aortic arch was anastomosed to the trimmed descending thoracic aorta in an end-to-side fashion (**c**)

**Fig. 3:**
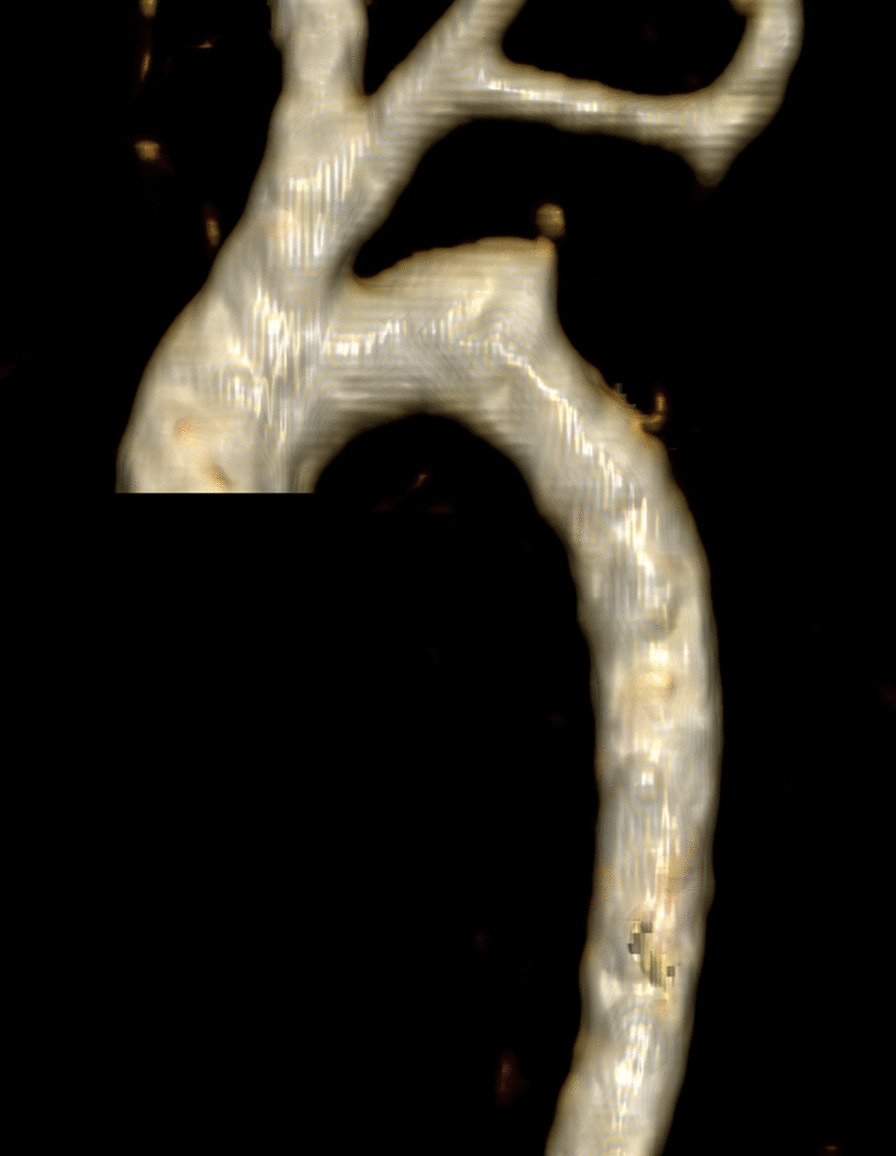
Postoperative three-dimensional computed tomography angiography showing a wide aortic arch without stenosis

The intraoperative findings revealed that the fourth aortic arch was connected to the descending aorta through a stenotic isthmic portion, and a stenotic area was also observed between the PFAA and the descending aorta (Fig. 2a). Cardiopulmonary bypass (CPB) was established by arterial cannulation of the innominate artery and bicaval venous cannulations. After cardiac arrest was induced, the connection between the fourth aortic arch and the descending aorta was divided. Antegrade cerebral perfusion was started under 25 ℃ of body temperature, and the CPB flow rate was 50 ml/min/kg. The PFAA was also divided, and the ductal tissue was resected completely from the descending aorta (Fig. 2b). The repair was performed in an end-to-side fashion between the proximal stump of the fifth aortic arch and the trimmed descending aorta (Fig. 2c). The ASD was closed primarily, and the CPB was weaned successfully. The perioperative period was uneventful. The patient was extubated three days after the operation. Postoperative echocardiography showed a wide aortic arch with improved left ventricular function and no mitral regurgitation. The postoperative three-dimensional CT angiography showed a wide aortic arch as well (Fig. 3). The patient was discharged from the hospital 18 days after the operation and followed up 25 months without complications (Additional file 1).

## Discussion and conclusions

In 1969, Van Praagh and Van Praagh first reported a PFAA in a male patient [7]. PFAA is usually located below the fourth aortic arch. As a result, the distal aortic arch takes a bifurcated shape, which is also called a double-lumen aortic arch or double-barreled aorta. Conventionally, mammals have six pairs of primitive pharyngeal arches, which form the aortic arch and the head and neck vessels. After normal development, the left fourth aortic arch develops as the normal left aortic arch, and both sixth aortic arches develop as the pulmonary arteries. Ductus arteriosus originates from the distal portion of the left sixth aortic arch. The fifth aortic arches are thought to be either absent in humans, or transient structures that leave no remnant in the definitive arch system. When the fifth aortic arch persists and forms an additional aortic arch, it results in PFAA without a known mechanism. In a review of the clinical practice of PFAA, PFAA was classified into four types according to the connection of the vessels and the direction of the blood flow: systemic-to-systemic, systemic-to-pulmonary, pulmonary-to-systemic, and bilateral types [8]. To be considered as a true fifth aortic arch, the channel must be confined to the extrapericardial space, must arise from the ascending aorta proximal to the origin of brachiocephalic arteries, and must terminate either in the dorsal aorta or in the pulmonary arteries via the persistently patent arterial duct. However, the true embryological origins of the structures described as PFAA remain controversial. There are possible alternative explanations such as the persistence of dorsal collateral channels or extensive remodeling of the remaining pharyngeal arch arteries [9].

A review of 26 cases of PFAA by Lambert et al. [4] found that 76% of the cases presented with systemic-to-systemic connections via the fifth aortic arch. Of them, 38% and 19% of the cases were associated with the aortic coarctation and interruption of the fourth aortic arch, respectively. Various surgical methods for the PFAA with aortic coarctation have been reported previously including reconstruction of the fourth aortic arch using PFAA after ligation of the ductus arteriosus[1], interposition of a Gore-Tex tube graft (W. L. Gore & Assoc, Flagstaff, AZ, USA) between the ascending and descending aorta [6], patch aortoplasty augmenting both the fourth and fifth aortic arches [6], side-to-side anastomosis of the left common carotid artery and left subclavian artery and patch augmentation of the coarcted segment [5], end-to-end anastomosis of the PFAA and descending aorta after making one aortic arch from the fourth and fifth aortic arches [2, 4], and end-to-end anastomosis between the fifth aortic arch and the descending aorta [3]. In our case, the aortic arch was reconstructed using end-to-side anastomosis between the fifth aortic arch and the descending aorta after complete resection of the ductal tissue (Fig. 2). There were several advantages to our strategy. First, because the anastomosis is simply performed between the fifth aortic arch and the descending aorta, this method is easy and effective. Second, this technique can be performed using the patient’s own tissue without any artificial patching material. Therefore, it has growth potential and no additional surgery is needed in the future even though the long-term follow-up of the patient is mandatory.

In conclusion, PFAA with aortic coarctation is a rare congenital cardiovascular malformation, which can be repaired by various surgical methods. Among them, our surgical treatment option without the use of any artificial material showed good postoperative results and some benefits.

## Supplementary Information

**Additonal file 1.** The figure shows a wide reconstructed fifth aortic arch connecting descending aorta and a patent original transverse aortic arch 25 months after the operation in follow-up echocardiography. DA = descending aorta; PFAA = persistent fifth aortic arch.

## Data Availability

The datasets used and/or analysed during the current study are available from the corresponding author on reasonable request.

## Abbreviations

ASD: Atrial septal defect
CPB: Cardiopulmonary bypass
CT: Computed tomography
DA: Descending aorta
LA: Left atrium
PFAA: Persistent fifth aortic arch
