# Supplementary figures and images for: Persistent fifth aortic arch associated with aortic coarctation: a case of surgical correction without artificial material
Source: J Cardiothorac Surg. 2021 Sep 28;16:281. doi: 10.1186/s13019-021-01664-y (PMC8480090; doi:10.1186/s13019-021-01664-y)

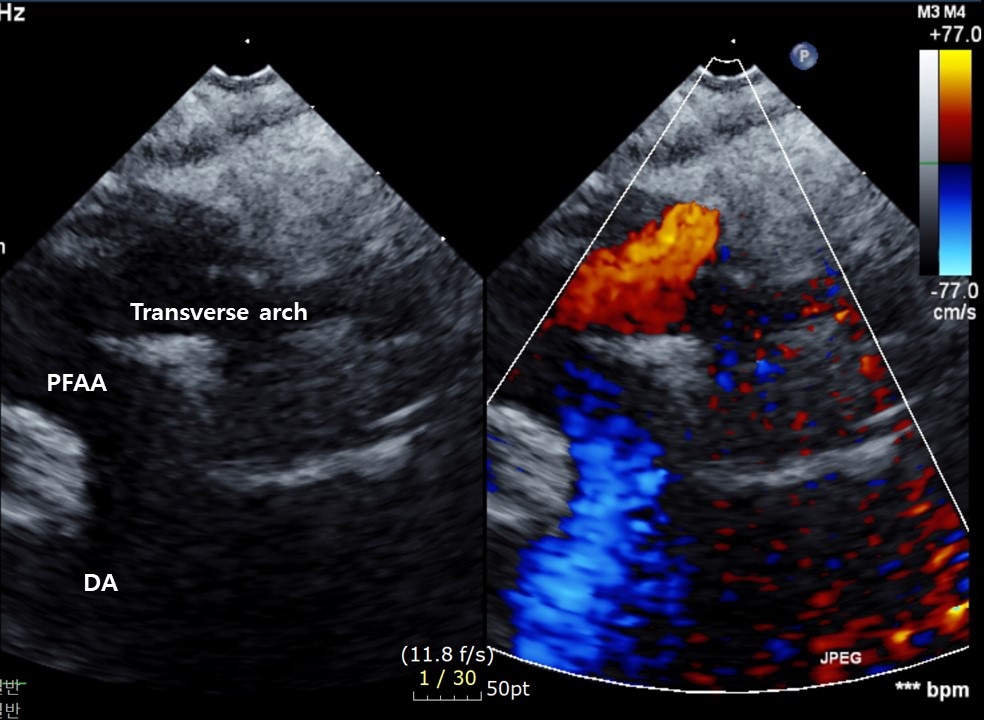

Supplement: Supplementary file 1 — Additonal file 1. The figure shows a wide reconstructed fifth aortic arch connecting descending aorta and a patent original transverse aortic arch 25 months after the operation in follow-up echocardiography. DA = descending aorta; PFAA = persistent fifth aortic arch. [file 13019_2021_1664_MOESM1_ESM.jpg]
